# Nursing students’ simulated home-visit learning experiences with dementia -a qualitative research

**DOI:** 10.1186/s12912-023-01232-w

**Published:** 2023-03-16

**Authors:** Youn-Joo Um

**Affiliations:** Department of Nursing, Dong-Yang University, 145 DongyangDaero, 36040 Punggi, Yeongju, Gyeongbuk Republic of Korea

**Keywords:** Simulation training, Qualitative research, Nursing student, Patient simulation

## Abstract

**Background:**

In response to the growing demand for community nursing, practical and dynamic changes in educational methods are essential to nurturing competent nurses. The aim of this study was to explore the learning experiences of nursing students’ simulation-based community visits and understand these experiences in detail.

**Methods:**

This study followed Colizzi’s phenomenological research method. Nineteen participants were divided into three teams and participated in focus group interviews. The research question was: “How was your experience with the simulated nursing home visit?”

**Results:**

Four essential themes were identified: “burden of community nursing simulation-based learning,” “solving the problems faced by patients with dementia through teamwork,” “home-visiting nursing skills learned through physical practice,” and “community nursing competency growth.”

**Conclusion:**

The study results provide a basis for developing a community nursing curriculum with effective evaluation and management of community nursing home-visit education using simulation.

## Background

Dementia is a disease that negatively affects the daily life and quality of life of the elderly due to the gradual occurrence of complex physical, mental, and behavioral symptoms due to cognitive decline [1]. Patients with dementia develop symptoms such as wandering, anxiety, depression, delusions, sleep disturbances, urinary problems, and memory loss.

According to the World Health Organization [2], the approximate number of people worldwide with dementia is 50 million and is expected to more than triple to 152 million by 2050. Additionally, 5–8% of the population over the age of 60 years suffers from dementia, and it is the fifth leading cause of death worldwide.

As the population with dementia gradually increases, the admission rate of old people with dementia or cognitive decline to senior medical welfare facilities tends to increase [3], but Korea lacks sufficient facilities for dementia patients [4]. Therefore, most dementia patients receive care at home from their families rather than from specialized institutions or facilities. Therefore, for those receiving care at home, it is important for experts to provide nursing care and management home visits according to the individual situations in each home and the patient’s case because each family has different challenges, situations, and environments [5].

Accordingly, in 2017 the Ministry of Health and Welfare introduced dementia as an issue of national responsibility rather than a problem of individuals and families and has established 256 dementia relief centers nationwide centered on the Central Dementia Center to strengthen support for dementia patients and their families [4]. With the announcement of the 4th Comprehensive Dementia Management Plan (2021–2025) in September 2021, various community resources centered on the National Dementia.

Support Center were linked to provide patients with specialized management according to their degree of dementia progression and suitable facilities or medical institutions as needed. The focus was on realizing a dementia-safe society where people can live with their families in the community [4].

Accordingly, the role of home-visiting nursing at the Dementia Support Center is to provide various health services such as dementia patient health care, dementia symptom management, medication management, health education, nursing, and the addressing of grievances. These services can provide nursing care tailored to the individual patient’s home situation and health condition, and are recognized as professional services that help patients live with their families at home without experiencing the difficulties associated with adapting to unfamiliar hospitals [6]. Additionally, through home-visiting nursing, the main caregiver of a family member with dementia often complains of health problems such as fatigue and disease along with life stressors such as restricted social activities and increased psychological burdens. From this perspective, caregiver education and training on health management methods suitable to the circumstances of the family members of patients with dementia, and education and training on stress management methods tailored to the home can also be provided by home-visiting nurses [7].

Home-visiting nurses play a role as a mediator in the relationship between health care professionals, patients, and caregivers in providing home-visiting nursing for patients with dementia, so high-level nursing skills are highly emphasized and necessary. Accordingly, home-visiting nurses are required to have the ability to communicate and cooperate with experts for consultation and diagnosis, preventive activities, and case management, as well as the ability to cooperate with dementia patients, their families, and the community [8]. However, due to COVID-19, opportunities for field practice among nursing students in home-visiting nursing for dementia patients have significantly decreased, and providing inexperienced nursing care to dementia patients can cause negative side effects, so sufficient prior education is needed. Therefore, education using simulation is required to develop nursing students’ home-visiting competency for caring for patients with dementia [9, 10].

Simulation-based learning is effective because it mimics actual clinical situations and practices in a safe environment that is tolerant of mistakes and allows practice and repetition [11]. Simulation-based home-visit nursing research involves directly analyzing various home environments through simulation to identify problems and nurture cultural competency to suit these environments [12]. The studies available on conducting home visits through simulation identify an improvement in the perception of self-efficacy, an increase in critical thinking [13, 14], emotional control skills, empowerment, and self-efficacy in nursing students when they develop this teaching methodology in a home visit environment [15]. Additionally, a simulation of receiving care support through Korea’s health insurance, etc., was conducted [16]. However, there is no study that has explored the simulation experience of nursing students targeting dementia patients, and it is necessary to explore nursing students’ experiences through this study.

This study is expected to contribute as literature for the development of practical nursing interventions that can enhance the coping ability and nursing competency of home-visiting nurses with patients with dementia by identifying nursing students’ experiences with home-visiting nursing simulation education for dementia patients and exploring their meaning.

It further aims to reveal the essence of home-visiting nursing simulation education for dementia patients by applying a phenomenological method to confirm the nature of human behavior and the meaning of experience derived from behavior. The study question is “what is the nursing student’s experience with home-visiting nursing simulation education for dementia patients?”

## Participants, ethics, and methods

### Research design

This qualitative study applied a phenomenological method to assess the value of home-visiting nursing simulation practice sessions undertaken by nursing students. The study’s purpose was to explore the essence of nursing students’ simulation-based community home-visiting nurse learning experiences and to understand associated phenomena. The research question was, “How was your experience with home-visiting nurse simulation-based learning as a nursing student?”

### Participants

The study participants were students enrolled in the 4th year of training in the Nursing Department at D University located in Y city and had already completed the home-visiting nursing simulation class. Among 61 4th-year nursing students, students who took the home-visiting nursing simulation class posted an article on the department’s community bulletin board about recruiting interested students for an interview. The purpose and method of the study were explained face-to-face to the 19 nursing students who expressed their interest in participating in the study, and ethical considerations were explained. Next, the nursing students who voluntarily expressed their intention to participate in the study completed a research consent form and scheduled a focus group interview. The selection of participants was conducted through convenience sampling until the data were saturated, and 19 people finally participated. There were no dropouts. The sociodemographic characteristics of the participants are shown in Table 1.

**Table 1:** General Characteristics of Participants in a Focus Group Interview (*n* = 19)

Characteristics	Categories	*n* (%)
Sex	Male	3 (15.8)
	Female	16 (84.2)
Age	20–25	13 (67.4)
	25–30	4 (21.0)
	≥ 30	2 (10.5)
Job	College student	19 (100.0)
Major	Nursing	19 (100.0)
Year	4th	19 (100.0)

### Data collection

Data collection occurred from November 18, 2021, to December 17, 2021. Research data were collected through face-to-face focus group interviews. Participants were interviewed by three focus groups of five to six people. This was based on the fact that the number of participants for focus group interviews is generally composed of three groups [17]. The qualifications of the researchers included those who had doctoral degrees in nursing and experience in conducting qualitative research. In this study, the professor in charge of the home-visiting nursing simulation class participated in the focus group interviews and analysis as a researcher who is female. The researcher had a relationship with the students through the major classes before the start of the research, so it was appropriate to conduct the interview. The researcher conducted qualitative research on nursing student education for many years, published several in top journals, and attended various qualitative research method workshops. The researcher worked as a school nurse for 12 years and is currently teaching nursing students at a university. The interview was conducted in a relaxed atmosphere at the university lecture hall that is frequently used by students. Participants fully understood the purpose of the study, participated voluntarily and were willing to share their experiences. The interviews were unstructured, which allows the researcher to interview the participant with a minimum of questions.

Participants were allowed to discuss their views freely and encouraged to give a full account of their experiences. Interviews continued until the participant’s story was repeated or the discussion produced no new stories. After the first interview was recorded, a second interview was conducted individually with 3 participants by phone to avoid incomplete interview content. After conducting 19 focus group interviews, the data were saturated and no further recruitment was conducted. The duration of one interview for each focus group was approximately 60 min. The interviews were recorded as they proceeded and transcribed by the person who conducted the interview.

### The home-visit nursing simulation process

This simulation program consisted of one 100-minute session. The session covered the scenario of a nurse home-visiting a dementia patient’s home to provide nursing care. The session consisted of pre-briefing (30 min), scenario (30 min), and debriefing (40 min) in this sequence. Scenarios were placed in simulated homes that replicated real-life situations, with 3–4 students attending the scenarios while others observed.

The first step, a pre-brief (30 min), introduced the students to learning objectives and the basic rules of respect and confidentiality, thus creating a safe learning environment. After a brief introduction of the scenario by the professor, the students decided in groups how to interact with the patient through discussion. The patient role was played by a student from another team.

During the second step, the scenario (30 min), rapport formation and assessment (home environment, patient symptoms, caregiver counseling), nursing planning and intervention (dementia symptom management education, caregiver education, and psychological support, guidance on how to take medications), and nursing evaluation (evaluation of educational content, greetings, and appointments for the next visit) were conducted.

During the third debriefing phase, the nursing students share their thoughts on the scenario.

Open-ended questions such as “What do you think happened to the patient?”, “What did you want to achieve?”, and “Tell me more about it” elicited information and allowed time for self-assessment.

After that, the session was finalized after receiving feedback from the professor and other nursing students.

### Data analysis

In this study, data collection and analysis were performed according to the analysis methods and procedures of Colaizzi [18], and data were analyzed using Microsoft Excel (Microsoft Corp., Redmond, WA, USA). As a result of the analysis, 252 codes, eight sub-categories and four categories were analyzed.

The method utilized was as follows. In Step 1, the overall meaning was grasped by reading and re-reading the transcribed content. In Step 2, the researcher reviewed the collected data and extracted the sections that were judged to have occurred in the process of remembering and representing the experiences of the research participants to extract meaningful statements from individually collected data. These extraction results were verified by three experienced phenomenological researchers to prevent ambiguous or unreasonable meanings in extracting meaningful statements. Through this, data were extracted by selecting meaningful sentences or phrases representing home-visit nursing simulation-based learning. In Step 3, while carefully examining the meaningful statements, redundant expressions were excluded, and general and abstract statements were constructed. In Step 4, the meanings that were constructed by the researcher were verified to ensure they fit the intentions. In Step 5, the statements were categorized according to the subject and essential themes were collected based on the intentions. In Step 6, the participants’ experiences were described according to the essential themes of the data analyzed to that point and the fundamental structure was stated. In Step 7, the credibility, transferability, dependability, and confirmability suggested by Guba and Lincoln [19].

] were identified to determine the study’s rigor.

For data collection credibility, open-ended questions were asked during interviews to allow participants to freely express their thoughts and experiences and to minimize midway intervention. The researcher recorded the interviews according to the focus group and transcribed the recorded material. Additionally, whether it was transcribed was checked by comparison with the recorded file, and the researcher conducted data analysis. In order to verify the credibility of the analysis, the primary and assistant moderators reviewed and discussed the similarities and differences of the data belonging to the codes and sub-categories for each stage of the analysis, and modified the name of categories. The content of the analysis and naming were reviewed by a professor with extensive experience in qualitative research. To ensure applicability, the results of the analysis were shown to another university student who had a similar experience but did not participate in the study and confirmed that it was meaningful and applicable in light of his own experience. In order to ensure auditability, the interview questions, progress, and analysis process were described in as much detail as possible. In order to maintain neutrality, during the interview it was emphasized that the researcher only fulfilled the role of the interviewer, and efforts were made to minimize and objectify the influence of the teacher-student relationship. The research director who conducted and analyzed the interview obtained a doctorate in qualitative research and is constantly active in qualitative research-related societies.

Additionally, he has conducted a number of qualitative studies, including focus group studies, targeting various participants such as community nursing, nursing students, nurses, and education.

### Ethical considerations

This study was approved by the bioethics committee of Dongyang University before data collection and was assigned ethical approval number 1041495-202202-HR-02-01 on November 17, 2021. The researchers wrote to the participants and shared the aims and methods of the research and explained that confidentiality was protected and participation in the research was completely voluntary. Informed consent was obtained from all participants included in the study. All the steps and methods were performed in accordance with the relevant guidelines and regulations. Additionally, the participants were informed that they could leave the research at any time without providing a reason. All procedures in the study were conducted according to the Declaration of Helsinki. Participants voluntarily participated. Confidentiality and anonymity were guaranteed, and the recordings were not used for any purpose other than the specified research. Participants were informed that the recorded files were password protected, not connected to the internet, and would be destroyed.

## Results

This study explored the experiences of 4th-year nursing students who participated in simulation-based home nursing home-visits and that had the meaning extracted. The phenomenological analysis method suggested by Colaizzi [18] was used for data analysis. A total of 252 meaningful statements were analyzed. Of these, eight sub-categories were chosen based on repeated or similar statements, then grouping and verification of comparable data, and four categories were derived (Table 2). The subcategories of this study comprised four subcategories: “burden of community nursing simulation-based learning,” “solving the problems faced by patients with dementia through teamwork,” “home-visiting nursing skills learned through practice,” and “growth in community nursing competency”. Finally, Nursing students’ simulated home-visit learning experiences were abstracted into one category, “cooperating and growing together in a simulated situation”.

**Table 2:** Nursing students’ simulated home-visit learning experiences

Categories	Subcategories
Burden of community nursing simulation-based learning	Fear of unfamiliar teaching methods
	Learned the difference from simple nursing skills practice
Solving the problems faced by patients with dementia through teamwork	Having fun in team class
	Interaction through collaboration
Home-visiting nursing skills learned through practice	Changed from a passive observer to an active nursing agent
	Motivate learning by acknowledging one’s shortcomings
Community nursing competency growth	Take responsibility as a professional
	Improving nursing confidence

### The burden of community nursing simulation-based learning

When nursing students encountered the new subject of simulation practice, they felt burdened about how the class was conducted and taught. They experienced the burden of classes in which communication skills and nursing skills were put into a room and nursing interventions were conducted according to the situation that was required at the moment.

#### Fear of unfamiliar teaching methods

The participants felt awkward and afraid about the simulation practice since it was their first time.

“It was my first time doing a simulation, so there was a lot of pressure and awkwardness to act with my classmates.” (Participant 10).

“I also doubted whether the nursing intervention was appropriate.” (Participant 3).

“Initially, it wasn’t easy when I realized that it was a class where practical situations were presented, and practice was conducted.” (Participant 14).

#### Learning the difference between previous nursing lessons and simple nursing skills in practice

Initially, there was a misunderstanding that the core nursing competency test was being repeated. However, after the simulation-based learning exercise began, the nursing students realized it differed from their traditional practice. Participants felt embarrassed because they had to virtually respond to unexpected situations in a given scenario.

“I thought the experience would only go according to the previous framework; however, I was embarrassed by the unexpected situation that was staged.” (Participant 15).

“When a dementia patient behaves unexpectedly, I can’t think of anything, so I don’t know how to respond or what kind of nursing care to provide. Although it was a simulation situation, the same atmosphere was as the actual clinical situation.” (Participant 10).

### Solving the problems faced by patients with dementia through teamwork

Because the simulation class is not an individual practice, but a team practice involving various participatory roles, the nursing students had not developed close relationships, but they developed positive teamwork by sharing their opinions in order to fulfill their roles well.

#### Having fun with teammates

The participants had the burden of performing the test alone; however, the team-based simulation practice, which consisted of four to five people, allowed them to have fun and reduced tension.

“It was fun to practice with the team members. It was awkward at first, but it was nice to get to know each other while doing the script.” (Participant 13).

“As we progressed as a team rather than alone, the burden of making mistakes was reduced, and as each role was designated, the burden was reduced, and we became mutually reliant on one another. I was in charge of educating the caregivers, and was able to complete it safely by dividing the responsibilities of patient medication education, vital sign measurement, and emergency response.” (Participant 16).

#### Interacting through collaboration

Participants were pleased that they could help each other and cooperate through teamwork and that team members could supplement their shortcomings. They believed that better nursing behaviors could be implemented when they collected various opinions among the team members through passionate discussion on nursing care activities for patients with dementia.

“Because we studied together with the team members, we could engage in simulation practice more passionately.” (Participant 3).

“While preparing with the team members, I was able to learn what I was lacking or not prepared for. It was nice to be able to choose better actions based on various opinions.” (Participant 7).

### Home-visiting nursing skills learned through practice

The Nursing students constantly discussed with team members and tried to get answers in the process of solving the problem of visiting nursing care scenarios for dementia patients. Their interest in nursing knowledge increased through recognition of the difference between the theoretical knowledge of visiting nursing for patients with dementia and the actual situation and learning about the application of the actual nursing process.

#### Changing from being a passive observer to an active nursing agent

While the simulation scenario was being implemented, the participants came to understand the patient’s nursing problems and considered the appropriate nursing intervention to be provided. In community-based nursing, nursing students only observe nurses providing care, and nurses mainly provided the care themselves. However, in the simulation-based learning experience, there was an opportunity to experience home-visit nursing practices because the participants had to judge and decide how the patient would be cared for on their own.

“During clinical practice, I went to a home-visit with a nurse at a public health center, but as a student, there was very little I could do, which was regretful. However, through this simulation-based learning, I was proud to have a more active experience with home-visit nursing.” (Participant 3).

“Through the simulation activity, I understood the patient better. There was even time for the caregiver to learn how to empathize and educate the patient.” (Participant 8).

“During clinical practice, I watched a nurse from an observational point of view. When I actually tried providing nursing care myself, I realized that, yes, this is home-visit nursing.” (Participant 6).

#### Learning is motivated by acknowledging one’s shortcomings

The participants felt their lack of nursing hands-on experience while participating in the simulation-based nursing scenario. They recognized that they could not easily answer the patient’s unexpected questions. Therefore, they learned that they should develop the ability to respond quickly based on thorough knowledge. Additionally, they felt that their nursing skills, including therapeutic communication, were lacking, and thought that they should correct this in the future.

“When a patient asks me a question, I think it is important to understand the patient’s situation and have accurate nursing information to answer the question properly.” (Participant 11).

“I don’t know how to empathize with a caregiver when they are having a hard time. I think I should study more about therapeutic communication.” (Participant 12).

“I think it was very helpful when the professor pointed out what was wrong and gave me feedback during the debriefing time to look back on the practice after finishing the simulation.” (Participant 5).

### Community nursing competency growth

Nursing students were proud to be able to deal with situations they hadn’t experienced in clinical practice as if they were real situations through participating in nursing simulation education for dementia patients and gained confidence in their ability to cope with situations that occur in actual nursing visits improved.

#### Take responsibility as a professional

Through simulation-based learning, the participants felt a sense of responsibility and mission to provide professional nursing care that truly helps patients. They felt that each nursing procedure they performed significantly impacted the patient. This helped them decide to develop their professionalism.

“To a nurse, any patient is just one of many patients, but from the patient’s point of view, they are unique. A sense of responsibility grew with the recognition that patients feel you are the only medical staff they can trust and rely on.” (Participant 15).

“I felt that the quality of nursing visits is determined by the visiting nurse. So I thought of studying to gain more specialized knowledge and skills to care for people with dementia and their families.” (Participant 2).

“When I approached the simulation as if it was a real situation, I felt the responsibility of the nurse position. Simultaneously, while dealing with patients and caregivers, I thought about how I would feel from the perspective of the patients and caregivers.” (Participant 3).

#### Improving nursing confidence

Participants thought that their ability to identify and cope with patient problems in various changing situations had improved through participation in this practical simulation-based learning.

“Contemplating what I did well during the debriefing class, I was able to gain a lot of confidence due to the generous praise of the professors and my classmates.” (Participant 3).

“In clinical practice, to be honest, as a nursing student, there was little I could do for myself. However, although it is standardized nursing practice for patients, I was very proud to be able to think and judge comprehensively for each situation and provide nursing care. Ah, I thought that this is how I was doing it, and I felt confident that I hadn’t felt previously in clinical practice.” (Participant 9).

## Discussion

In this study, in order to understand the educational experience of 4th year nursing students in a visiting nursing simulation for dementia patients, the meaning and nature of the educational simulation experience were described by applying a phenomenological method. Through focus group interviews, the results of this study derived experiences related to the “burden of community nursing simulation-based learning,” “solving the problems faced by patients with dementia through teamwork,” “home-visiting nursing skills learned through physical practice,” and “growth in community nursing competency”.

Nursing students’ experiences of home-visiting nursing practice education for patients with dementia are as follows. Although the researcher provided information on how to proceed with the class before it started, the nursing students did not fully understand the simulation education initially. Additionally, they were confused because they knew that the class was solving problems on their own without an established protocol and had to interact with colleagues whom they were not familiar with. Furthermore, they complained about the burden of performing post-surgery in response to the sudden situations of dementia patients. This is a similar result to those of previous studies [20, 21] that applied team-based simulation to nursing students, and it is thought that this is because students who are accustomed to lecture-style classes feel burdened by classes that they conduct more independently. Practical training in home-visiting nursing care for patients with dementia is difficult to learn and apply in a short time [22]. Therefore, to reduce the students’ burden from the home-visiting nursing simulation classes for patients with dementia, it is necessary to participate in this type of learning several times before class and to promote interaction among classmates by sharing opinions and feelings with team members. As the class progressed, the students were able to overcome the burden of the new class method and environment that they had initially and were able to confirm their theoretical knowledge once again by sharing opinions with each other through teamwork. Also, by realizing the necessity and effectiveness of teamwork, positive teamwork developed and the students became immersed in the dementia patient simulation. Akaike, et al. [23] stated that in the case of simulation training, it is necessary to understand the limits of teamwork, communication, and simulation situations, as well as the learners’ practical skills.

In order to relieve the burden of the primary class method and utilize the simulation learning method more efficiently and effectively, it is necessary for students to familiarize themselves with the simulation class method before the class begins and form teams by sharing opinions with team members. Additionally, nursing students shared their feelings after the simulation class, reanalyzed the contents of practice, and exchanged opinions with the instructor and students. Through this, new knowledge was obtained, and the relationship between team members and other teams’ practices was reviewed to improve situational nursing skills and capabilities. In particular, becoming more motivated resulted from comparing one’s own performance with their team or other team students, which is a similar result as seen in previous studies [24, 25]. It is thought that nursing students formed a cooperative learning structure with peers and a positive attitude among learners rather than one-sided knowledge transfer through simulation education. The participating nursing students realized the importance of communication with patients and recognized the problems and reactions of patients through the simulation class. Additionally, it provided an opportunity to apply clinical nursing knowledge that they knew theoretically, and their confidence in performing nursing visits for patients with dementia improved.

These findings are consistent with previous studies that showed that nursing skills and team collaboration abilities were improved after patient simulation education [22, 26, 27]. In particular, this study supports it as an effective educational method to increase confidence in nursing practice, and it became an opportunity to feel a sense of responsibility through the experience of directly affecting the health of patients through their nursing. Previous studies have shown that nursing students realize their lack of professional knowledge and preparation through simulation-based learning [28, 29] and improve their confidence in nursing practice [30]. Therefore, the practice education of home-visiting nursing care for dementia patients will supplement the limitations of limited education in clinical practice and contribute to improving practical clinical performance.

Additionally, as the number of dementia patients increases and care becomes more important in the community, home-visiting nursing simulation education applies more clinical expertise and skills to nursing students, and practices problem-solving processes by situation considering the patient’s environment and available resources. It is thought that this will be helpful in the nursing practice of future nurses.

This study has some limitations including that it analyzed data from only a small number of participants from one university, so it is difficult to generalize and validate the results. Additionally, this study was conducted targeting students who were unfamiliar with the simulation class method in order to reduce biases based on existing simulation education experience. In this process, the first simulation experience may have been biased rather than the nursing home-visiting simulation experience.

However, this study presented specific and realistic educational experiences of nursing students who completed home-visiting nursing simulation education for patients with dementia. Furthermore, it provides meaningful basic data for grasping the reality of home-visiting nursing education for patients with dementia and for practical application and dissemination.

## Conclusion

Simulation classes are offered as an educational and innovative tool that favors home learning for nursing students. The use of simulation brought positive benefits to nursing students through improved self-confidence, nursing skills, communication skills, and reflective thinking. Based on this information, simulation should be considered as an instructional methodology in university education programs in community nursing subjects. This will improve clinical practice by facilitating the training of future medical professionals. Despite these promising results, further research is needed in this area to evaluate the development of simulation training scenarios for dementia prevention, simulation facilitator training, and other training using simulation methods.

## Data Availability

The datasets generated and/or analyzed during the current study are not publicly available because the data is part of an unpublished dissertation and the data is in Korean but are available from the corresponding author upon reasonable request.
